# Progressive Medicine, Vol. III, September, 1900

**Published:** 1901-05

**Authors:** 


Progressive Medicine, Vol. III, September, 1900.—A quarterly digest of advances, discoveries and improvements in the medical and surgical sciences. Edited by Hobart Amory Hare, M. D., Professor of Therapeutics and Materia Medica in Jefferson Medical College of Philadelphia. Octavo, handsomely bound in cloth, 408 pages, with 14 engravings. Lea Brothers & Co., Philadelphia and New York. Issued quarterly. Price, $10.00 per year.

One will not be disappointed in Progressive Medicine. So far the writer of this review has never failed to find something rich in each volume examined. The present volume begins with a most admirable article on "Diseases of the Thorax and its Viscera," by Dr. William Ewart, of London. In this article is set forth all of the advances made during the past twelve months.

The next chapter is devoted to "Diseases of the Skin," by Henry W. Stelwagon, M. D., of Philadelphia. This article is particularly interesting on account of the advances in therapeutics as applied to diseases of the skin.

Dr. William G. Spiller, Professor of Diseases of the Nervous System in the Philadelphia Polyclinic, has a most excellent review of recent literature. The subjects receiving most attention are uræmic hemoplegia, post-anesthetic paralysis, cerebro-spinal meningitis, anesthesia by intra-spinal injections of cocainetic douloureux and its treatment by arsenic acid and by extirpation of the Gasserian ganglion, etc.

The article upon the subject of obstetrics is contributed by Dr. Richard C. Norris, of the Pennsylvania University, and it fully brings out all of the advances in that science during the past year.

